# Incorporating Patient Needs and Perspectives in Additional Risk Minimization Measures and Other Pharmacovigilance Deliverables - A Framework and Implementation Roadmap

**DOI:** 10.1007/s43441-025-00844-5

**Published:** 2025-07-25

**Authors:** Linda Smeding, Robert Massouh, Farai Moyo, Marilyn Metcalf, Shannon Altimari, Ekaterina Edle von Dall’Armi, Elisa Formenti

**Affiliations:** 1 GSK, Avenue Fleming 20, Wavre, 1300 Belgium; 2 GSK, London, UK; 3 GSK, Philadelphia, USA; 4 GSK, Baar, Switzerland; 5 GSK, Munich, Germany; 6 GSK, Verona, Italy

**Keywords:** Patient centricity, Patient engagement, Pharmacovigilance, Risk minimization, Framework

## Abstract

Various initiatives and guidelines exist to support patient engagement (PE) throughout the lifecycle of a medicinal product. While the recent European Medicines Agency guideline on good pharmacovigilance practices (Module XVI; Revision 3) reinforces the importance of involving patients to create effective risk minimization strategies, frameworks supporting the systematic adoption of PE by Marketing Authorization Holders (MAHs) across pharmacovigilance, including the risk management system, are lacking. Furthermore, little is presented on the impact of patient review of additional risk minimization measures materials. We present a tested Pharmacovigilance Patient Centricity Framework describing key focus areas that can create the necessary infrastructure for systematic PE in effective risk minimization materials. Implementation of this framework highlighted the importance of collaboration to drive PE across the company at both local and global level, and externally, as relationships are established with patient organizations and best practices are shared with other MAHs. Therefore, this framework can be considered by other companies as a basis for developing a patient-centric approach to integrate the patient’s voice into pharmacovigilance deliverables.

## Introduction

Patient centricity is an essential concept in pharmaceutical research and development, extending beyond participation in clinical trials, throughout the lifecycle of a medicinal product [1–6]. Patients’ experiences of living with their disease and their unmet needs appropriately shape the balance of benefits versus risks associated with treatments [3, 4, 7–10]. In pre-clinical and early clinical phases of drug development, patients can provide significant insights into unmet medical needs, research priorities and endpoints, study design, and patient priorities [3, 11–15]. Integrating patients’ perspectives in clinical development increases the likelihood of successful safety monitoring and compliance. In Phase II–III studies, patients’ perspective can be considered to inform the safety guidance for clinical trials and their ethical review, thresholds for pausing or stopping studies, and the appropriate endpoints for regulatory approval. Furthermore, patients can review risk minimization documents to be used in drug development (e.g., safety aspects of the informed consent forms) and support the preparation of post-marketing educational programs. Once medicines are approved, patients’ lived experiences provide real-world evidence of longer-term effectiveness and safety [11, 12, 14, 16–19]. The post-marketing lifecycle management period is generally the time when rare events can emerge with broader and long-term use of medicines. Patients’ insights around these events and the appropriate communication of safety updates are natural and much-needed extensions of patient engagement (PE) in the ongoing safe use of medicines.

**Fig. 1:**
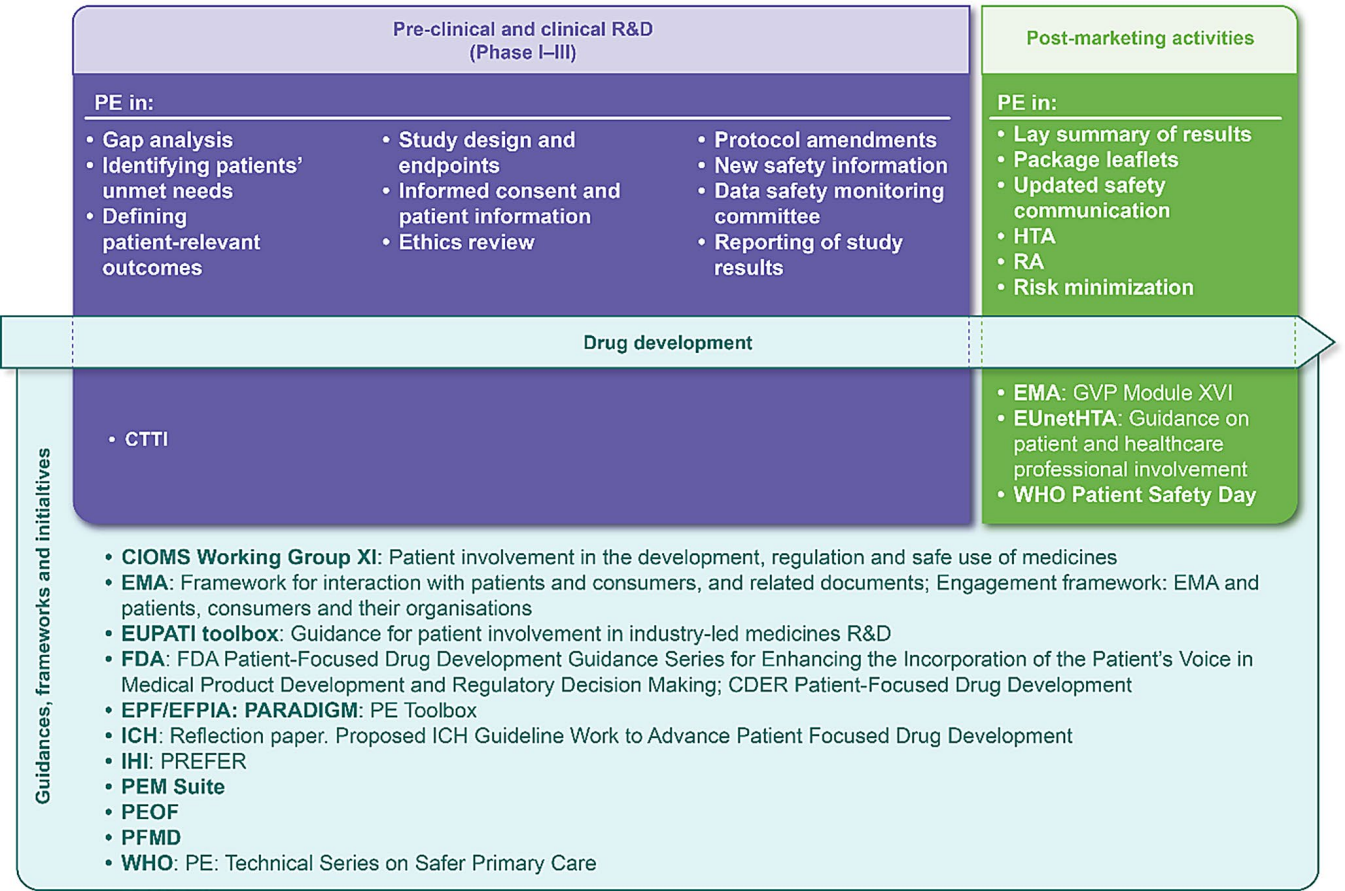
Drug development timeline with key areas of PE and the main guidances, frameworks and initiatives (non-exhaustive) integrating PE across the product lifecycle. CIOMS, Council for International Organizations of Medical Sciences; CDER, Center for Drug Evaluation and Research; CTTI, Clinical Trials Transformation Initiative; EFPIA, European Federation of Pharmaceutical Industries and Associations; EMA, European Medicines Agency; EPF, European Patient Forum; EUnetHTA, European network for Health Technology Assessment; EUPATI, European Patients’ Academy on Therapeutic Innovation; FDA, Food and Drug Administration; GVP, good pharmacovigilance practices; HTA, Health Technology Assessment; ICH, International Council for Harmonisation of Technical Requirements for Pharmaceuticals for Human Use; IHI, Innovative Health Initiative; PARADIGM, Patients Active in Research and Dialogues for an Improved Generation of Medicines; PE, patient engagement; PEM, Patient Engagement Management; PEOF, Patient Engagement Open Forum; PFMD, Patient-Focused Medicines Development; PREFER, Patient Preferences in benefit risk assessments during the drug life cycle; RA, Regulatory Affairs; R&D, Research and Development; WHO, World Health Organization

Various initiatives have been launched to involve patients across the product lifecycle, with regulatory authorities increasingly integrating the patient’s voice into their policies and recommendations (Fig. 1) [11, 19–32]. These initiatives consider some of the challenges of PE during drug development [11, 16–19, 33], which are also germane in post-marketing phase, such as:

- Communicating in patient-friendly but not condescending language.
- Inclusion of representative patients and patient organizations (POs) to provide informative perspectives for the patient populations of interest, including surrogates for patients who are very young, non-verbal, cognitively impaired, or otherwise in need of representation by an appropriate ally.
- Use of emerging technologies to provide broader access to medicines and communications without excluding populations who may already be underserved.
- Alliance with POs to support patients’ needs without overburdening the organizations.
- Support for patient participation that recognizes the value of their time and contribution without coercing their involvement.

Conceptual frameworks and roadmaps providing guidance on patient centricity in pharmaceutical research have been published to facilitate consistent PE [3, 4, 15, 34–40]. These frameworks and roadmaps have extended to support PE in pharmacovigilance [40–43]. One such roadmap describes a pragmatic approach for pharmacovigilance departments within the pharmaceutical industry, highlighting three hallmarks crucial for patient-centered safety departments: (1) adoption of a company-wide patient-centered culture supported by governance structure and ongoing training; (2) development of a PE framework, both internally and externally, through promoting PE with patient communities; (3) acquiring patient-centered competencies, such as increasing patient access to understandable information, e.g., labelling and other patient-targeted pharmacovigilance materials [43]. Additionally, the International Society of Pharmacovigilance has recently established the PE Special Interest Group to develop and promote PE in pharmacovigilance through various research and training activities, as well as publications, to raise awareness of the importance of PE in safety in collaboration with patients and POs, regulatory authorities, and other relevant stakeholders [40]. Various regulatory guidances exist to support the incorporation of the patient’s perspective specifically into pharmacovigilance activities (Table 1).

**Table 1:** Examples of key initiatives and guidances incorporating patient voice in pharmaceutical drug safety and pharmacovigilance

PV activity/deliverable	Initiative/Organization	Title	Objectives/key point	Reference
ADR reporting	European Parliament and Council of the European Union	Regulation (EU) 1235/2010 amending, as regards pharmacovigilance of medicinal products for human use, regulation (EC) No. 726/2004 laying down Community procedures for the authorisation and supervision of medicinal products for human and veterinary use and establishing a European Medicines Agency and Regulation (EC) 1394/2007 on advanced therapy medicinal products	Implementation of EU pharmacovigilance legislation 2012 strengthening the monitoring of EU-marketed medicines and expanding direct patient reporting of suspected ADRs throughout the EU	[47]
	European Medicines Agency	Guideline on good pharmacovigilance practices (GVP) Module VI – Collection, management and submission of reports of suspected adverse reactions to medicinal products (Rev 2)	Updated guidance on the collection, data management and submission of individual reports of suspected adverse reactions	[48]
	Innovative Health Initiative	WEB-RADR 2: Recognising Adverse Drug Reactions	Mobile application allowing patients to report ADRs directly to regulatory authorities, and to receive new alerts and up-to-date information on drugs	[49]
Benefit–risk assessment	Food and Drug Administration	Patient Preference Information - Voluntary Submission, Review in Premarket Approval Applications, Humanitarian Device Exemption Applications, and De Novo Requests, and Inclusion in Decision Summaries and Device Labeling Guidance for Industry, Food and Drug Administration Staff, and Other Stakeholders	Guidance on incorporating the patient perspective into benefit-risk profile of certain medical devices	[50]
	International Conference on Harmonisation	ICH Harmonised Guideline: Revision of M4E Guideline on Enhancing the Format and Structure of Benefit-Risk Information in ICH Efficacy - M4E(R2)	Guidance specifying inclusion of patient perspective in the Clinical Overview Section regarding therapeutic context, benefits, risks, and the benefit-risk assessment for marketing application	[51]
	Innovative Health Initiative	PREFER: Patient Preferences in benefit risk assessments during the drug life cycle	Project providing an overview on when and how patient preferences on benefits and risks should be incorporated into decisions-making throughout the life cycle of a medicinal products	[52]
	Council for International Organizations of Medical Sciences (CIOMS) XII	Benefit-risk balance for medicinal products	Report describing a lifecycle approach to developing a structured benefit-risk framework, incorporating the role of the patient (and patient experience data)	[53]
Risk management, risk minimization and risk communication	European Medicines Agency	Good practice guide on recording, coding, reporting and assessment of medication errors	Pharmacovigilance legislation promoting collaboration with patient organizations to minimize preventable harm from medication errors	[54]
		Guideline on good pharmacovigilance practices (GVP) Module XV – Safety communication (Revision 1)	Guidance outlining the importance of incorporating patients’ input in preparation of safety communications, including lay language materials (Sections XV.B.2. Principles of safety communication and XV.B.5.3. Documents in lay language to patients and the general public)	[55]
		Guideline on good pharmacovigilance practices (GVP) Module XVI – Risk minimization measures (Revision 3)	Guidance outlining the importance of patients’ input across all stages of RMM design and implementation (Section XVI.B.1.4. Engagement of patients and healthcare professionals in risk minimization)	[44]
	Council for International Organizations of Medical Sciences	Practical Approaches to Risk Minimization for Medicinal Products: Report of CIOMS Working Group IX	Guidance recommending inclusion of patients in risk minimization planning, implementation, and evaluation	[56]

ADR, adverse drug reaction; EC, European Commission; EU, European Union; PV, pharmacovigilance; RMM, risk minimization measure

Regarding risk management, the recently effective European Medicines Agency (EMA) guideline on good pharmacovigilance practices (GVP) Module XVI (Revision 3) [44] and the United States (US) Food and Drug Administration (FDA) risk evaluation and mitigation strategy (REMS) draft Logic Model [45] have further reinforced the importance of patient-centered healthcare involving various stakeholders (including patients) to create effective risk minimization strategies. These guidelines describe the opportunity to engage with patients across all stages of risk minimization design, implementation, and evaluation, to support appropriate adoption and maintenance of the risk minimization recommendations in medical practice.

While the guidelines re-affirm the importance of PE in risk management, to our knowledge, frameworks supporting the systematic adoption of PE by Marketing Authorization Holders (MAHs) across the risk management system are limited. Furthermore, there is limited guidance to support balancing value with timely PE in pharmacovigilance deliverables across the product lifecycle. Thus, PE may occur ad hoc and in a reactive manner, and in some instances be considered merely symbolic or tokenistic [46], with limited impact on the effectiveness of the risk management activities. Assessing the impact of PE in risk minimization activities is likewise challenging due to this lack of a systematic approach for such engagement and the limited published data describing PE outcomes. According to the available literature, to date, only one study reported proposals for engaging patients in risk minimization; this study revealed gaps in stakeholder input and research related to critical elements of implementation of risk minimization measures (RMMs), and indicated the need for an improved PE in RMM decision making [41]. However, to date, there is no study published on the impact of patient review of RMM materials.

**Fig. 2:**
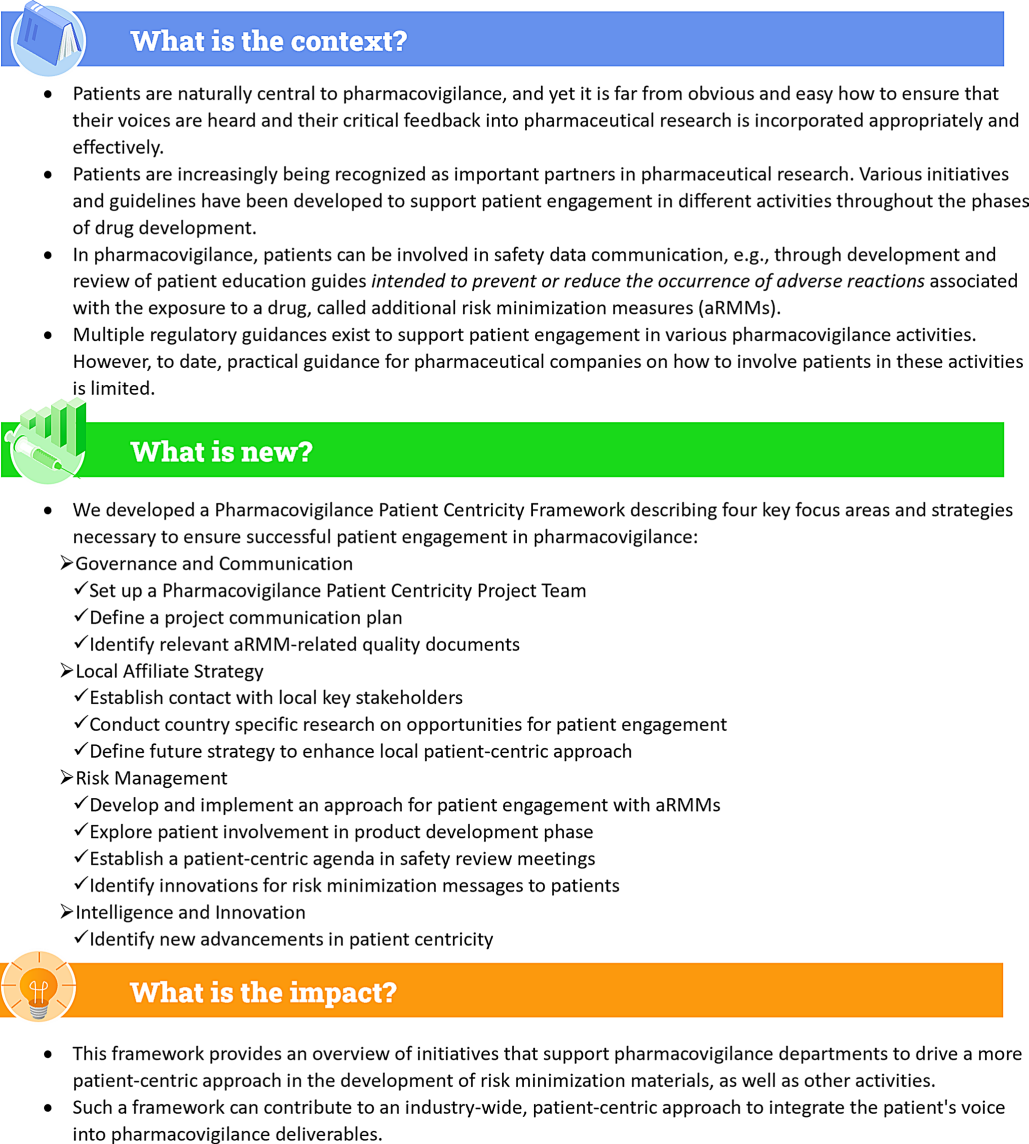
Plain language summary

To support MAHs to leverage a systematic and practical approach for PE across the pharmacovigilance system, including risk management, we propose a framework for consideration and adaptation according to company-specific needs. A plain language summary describing this framework is shown in Fig. 2.

## Context

In 2021, supported by the EMA draft GVP XVI Revision 3 guidance [44] (under public consultation at that time), a PE pilot project with a PO was initiated in a local pharmacovigilance department to test patient additional RMMs (aRMMs) for a hematology asset.

An advisory board of eight PO representatives was convened to review the patient educational materials to ensure these materials addressed key questions from patients, were understandable, and conveyed a clear and concise narrative. The feedback received in the PE pilot led to positive changes to the aRMMs in terms of design and messaging which were considered in a future update to the materials.

The pilot’s success highlighted the importance of PE in aRMM design. It also emphasized the need for a consistent and centralized framework across global and local departments to proactively integrate patient perspectives into aRMMs. As a result, a Pharmacovigilance Patient Centricity (PPC) group was established, taking learnings from the pilot, feedback received from other MAHs through collaborative platforms, as well as PE expertise from published guidelines and the literature to support the systematic adoption of PE in pharmacovigilance.

## Pharmacovigilance Patient Centricity Framework

**Fig. 3:**
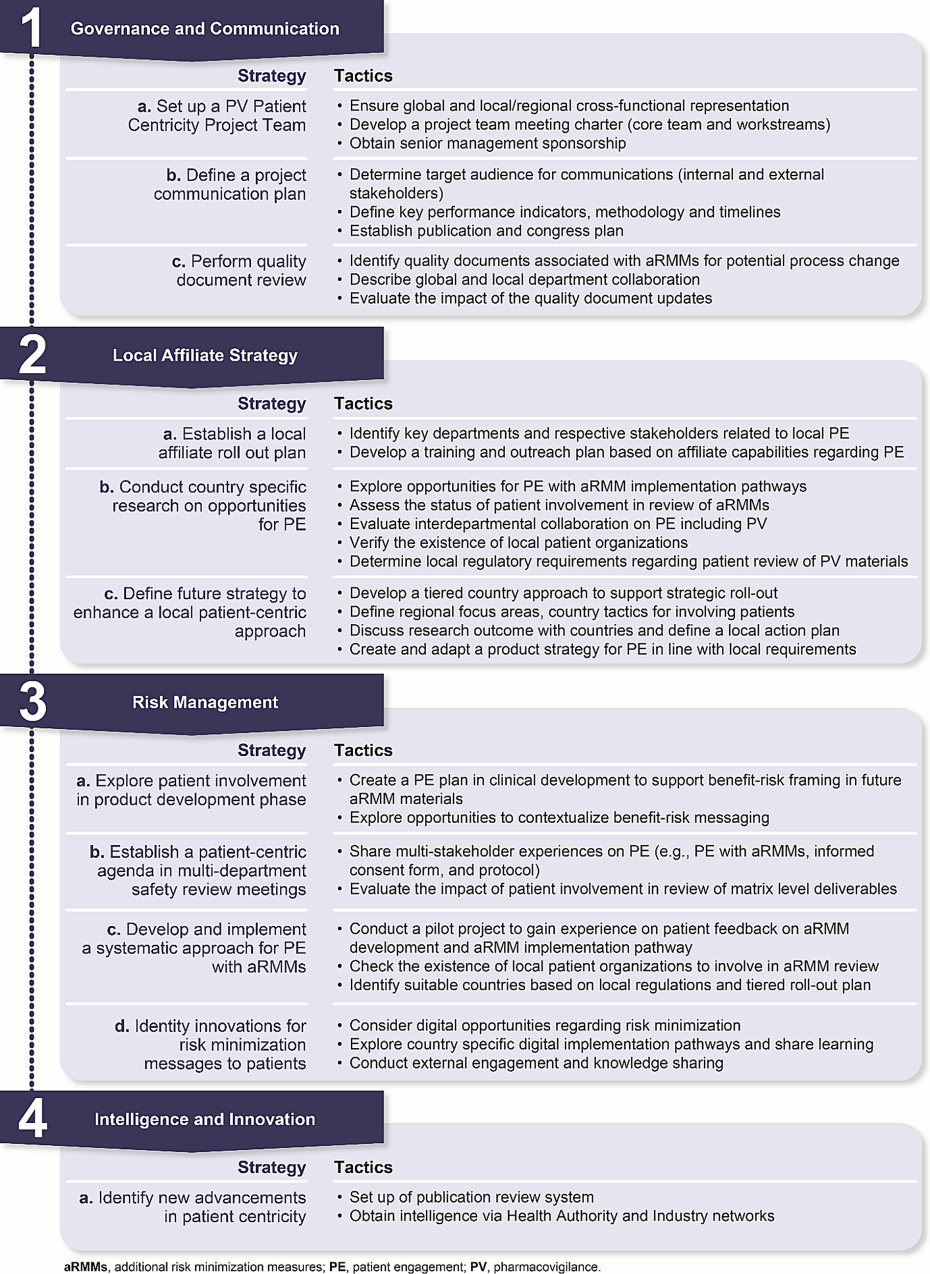
Framework for patient-centric initiatives in pharmacovigilance

The initial objective of the PPC group was to formalize PE in internal quality documents. Consequently, it became evident that more guidance and structure was needed to integrate PE in pharmacovigilance across the product lifecycle and to support cross-functional and global-local collaboration. As a result, the PPC Framework was developed and rolled out. The framework describes four focus areas, with specific activities to support delivery (Fig. 3).

## Key Learnings

### Global-local Collaboration

It is critical to ensure cross-functional stakeholders from both global and local functions are involved and contribute to these initiatives, as both global-focused (e.g., communication and engagement, intelligence and innovation, and development of a global risk management strategy) and local-focused strategies (exploring local regulations, capabilities for PE, and availability of POs) should be aligned.

### Explore Country-Specific Requirements for PE

To determine the opportunities for PE and to understand the impact PE may have at a country level, a questionnaire was circulated to local pharmacovigilance departments to determine the structure of their internal collaboration, retrieve intelligence on regulatory requirements, and determine opportunities to involve POs in aRMM/pharmacovigilance deliverables. The results highlighted variable opportunities for PE (for example, due to limited acceptable pathways for PE) and served as an important starting point to define future country-based strategies and measure progress in PE capabilities. Since the regulatory landscape regarding PE in aRMMs is evolving, the PPC Framework supports ongoing regulatory intelligence to continuously evaluate new opportunities in PE.

### Education and Adaptation

Implementation of a patient-centric pharmacovigilance framework requires continuous learning and adaptation, driven by adoption by the affiliates, updates to regulatory guidelines, negotiations with health authorities, best practices from other MAHs, and feedback received from patients and POs. Regular communication and best practice sharing across the company is critical to support the systematic uptake of this initiative. Beyond internal communication, external engagement is crucial to support knowledge sharing and pressure testing of the framework to ensure patient-centric approaches are maximized.

### Define the Strategic Approach to PE

Determining the need, value, and opportunity for PE in pharmacovigilance should be done as early as possible; from an aRMM perspective, considerations for PE should be made as soon as it is determined that routine RMMs are not sufficient to mitigate a risk associated with the product. Certain aRMMs (i.e., for first-in-class products) may need specific types of PE, with differing objectives to inform the broader messaging, the potential implementation pathways and/or measure their effectiveness. Thus, it is critical for the global and local functions of the MAH to be aware and involved in developing the strategy for PE.

There are different pathways regarding an MAH’s PE structure and communication channel with POs (approaches may be adaptable based on country regulations on MAH engagement with POs). MAHs may consider outsourcing to a third party or encourage engagement of the local pharmacovigilance departments directly with POs. Regardless of the pathways considered, it is important to communicate the specific objectives and outcomes of the PE as early as possible.

Sharing knowledge and best practices on patient centricity in pharmacovigilance and pathways for PE through dedicated benchmarking and collaborative platforms is crucial to drive consistency and accelerate progress towards patient-centric pharmacovigilance across industries.

### Potential Challenges in PE

The PPC Framework builds on the principles of the published guidelines (see Fig. 1), to ensure patients’ valuable time and efforts are respected and their insights are appropriately incorporated. Therefore, it is of great importance that any consideration for PE is driven by a project with a well-defined set of objectives that minimize the potential for ‘tokenism’ in PE. Several principles should be considered when planning for PE, including:

- Ensuring information collected is focused on the needs of patients and improved safety in the context of benefit.
- Using language that makes the project transparent for the participants.
- Using methodology that is accessible for participants representative of the target population to provide informed consent and participate meaningfully in the engagement.
- Posing insightful questions to understand the concerns of patients.
- Allowing adequate time to implement recommended changes.
- Implementing a robust feedback loop to share updates to patients on regulatory interactions and success/challenges in implementation of the PE recommendations.

## Future Vision

The proposed framework allows pharmacovigilance departments to implement actions to drive a patient-centric approach to RM and other pharmacovigilance activities. While the PPC Framework has initially centered around aRMM planning and design, more emphasis will be added on working with POs to support evaluation of effectiveness of the aRMMs, to leverage qualitative and quantitative methods to inform effectiveness of both risk minimization messaging and the tools.

The PPC Framework will continue to integrate measurements of success of the PE to determine the impact of the PE on product-specific deliverables. A centralized effectiveness checklist can support assessment of PE against specific intended outcomes. The checklist can be mapped out against data points for collection, including qualitative and quantitative analysis of patient/PO feedback and its generalizability, and the Regulatory Authorities’ feedback on the patient-driven proposals.

The PPC Team will continue to leverage the learnings from the initial aRMM-focused activities and expand these to other pharmacovigilance deliverables, including adverse drug reaction (ADR) reporting, pharmacovigilance sections of labels, and public summaries of pharmacovigilance deliverables, such as risk management plans (RMPs).

## Conclusion

Through the creation of a global framework for PE, our aim is to present an industry-wide, patient-centric approach that can continue to evolve to integrate the patient’s voice into pharmacovigilance deliverables.

The EMA GVP Module XVI (Revision 3) [44] and the US FDA draft REMS Logic Model [45] highlight the importance of appropriately designed RMMs that consider various elements of implementation science to create effective risk minimization strategies that can be integrated into medical practice, including a critical role of engaging patients in all aspects of RMM design, implementation, and evaluation. Next to RMMs, other pharmacovigilance deliverables (e.g., ADR reporting, product labelling, or RMPs) can benefit from a patient-centric approach.

To support this, we present a PPC Framework describing key focus areas that can create the necessary infrastructure for systematic PE to create effective risk minimization materials. This dynamic framework can be leveraged and adapted by other MAHs with the opportunity to extend PE to other pharmacovigilance deliverables, beyond risk minimization.

## Data Availability

No datasets were generated or analysed during the current study.
